# Simultaneous knockout of multiple *eukaryotic translation initiation factor 4E* genes confers durable and broad-spectrum resistance to potyviruses in tobacco

**DOI:** 10.1007/s42994-025-00216-5

**Published:** 2025-05-05

**Authors:** Yong Liu, Shuo Wang, Danyang Zhao, Chenglu Zhao, Haiqin Yu, Jianmin Zeng, Zhijun Tong, Cheng Yuan, Zhenghe Li, Changjun Huang

**Affiliations:** 1 Yunnan Academy of Tobacco Agricultural Sciences, Key Laboratory of Tobacco Biotechnological Breeding, National Tobacco Genetic Engineering Research Center, Kunming, 650021 China; 2 State Key Laboratory of Rice Biology, Institute of Biotechnology, Zhejiang University, Hangzhou, 310058 China; 3 Ministry of Agriculture Key Laboratory of Molecular Biology of Crop Pathogens and Insect Pests, Zhejiang University, Hangzhou, 310058 China; 4 Key Laboratory of Biology of Crop Pathogens and Insects of Zhejiang Province, Zhejiang University, Hangzhou, 310058 China

**Keywords:** Eukaryotic translation initiation factor 4E, Recessive resistance, Potyvirus, Gene editing, Durable resistance, Broad-spectrum resistance

## Abstract

Recessive resistance mediated by mutations in the eukaryotic translation initiation factor 4E (eIF4E), has proven effective against diverse potyviruses and is extensively utilized in breeding programs. However, the rise of resistance-breaking (RB) strains and emerging potyviral species necessitates the development of more durable and broad-spectrum resistance strategies. In this study, our field survey in Yunnan, China, identified potato virus Y (PVY) RB isolates, as well as the prevalence of tobacco vein banding mosaic virus (TVBMV) and chilli veinal mottle virus (ChiVMV), in tobacco carrying the recessive *va* locus, which lacks the *eIF4E1-S* susceptibility gene, due to a chromosomal deletion. Protein interaction and viral infection assays demonstrated that both eIF4E1-S and eIFiso4E-T are used by PVY RB as susceptibility factors for infection, with the combined inactivation of these genes confering durable resistance. Similarly, the knockout of *eIFiso4E-S,* in the *va* genetic background, provided effective resistance to TVBMV and reduced susceptibility to ChiVMV. Notably, pyramiding mutations in *eIFiso4E-S* and *eIFiso4E-T*, in *va* tobacco, generated plants exhibiting robust, broad-spectrum resistance, to all three viruses, without compromising plant development. These findings underscore the potential of stacking eIF4E mutations to engineer durable, broad-spectrum resistance to potyviruses in tobacco, offering a promising strategy for crop improvement.

**Supplementary Information:**

The online version contains supplementary material available at 10.1007/s42994-025-00216-5.

## Introduction

Potyviruses are the largest group of plant-infecting RNA viruses that cause significant crop losses worldwide (Yang et al. 2021). The potyvirus genome is a positive-sense single-stranded RNA (~ 10 kb) that is characterized by a viral genome-linked protein (VPg) covalently attached to its 5' end and a poly(A) tail at the 3' end. Unlike host mRNAs, potyviral RNA lacks a conventional 5' cap structure. Instead, the VPg protein acts as a functional mimic of the cap structure by specifically interacting with host eukaryotic translation initiation factors (eIFs), such as eIF4E or its isoform, eIFiso4E. This interaction enables potyviral RNA to hijack the host’s translation machinery, ensuring efficient viral protein synthesis and subsequent infection processes (Truniger and Aranda 2009; Wang and Krishnaswamy 2012; Wang 2015).

Members of the eIF4E family have been widely recognized as susceptibility factors for potyvirus infections. Loss-of-function mutations in these genes confer genetic resistance through a mechanism termed recessive resistance or loss of susceptibility (Hashimoto et al. 2016; Zlobin and Taranov 2023). This phenomenon was first demonstrated in *Arabidopsis* mutants, where specific mutations in *eIFiso4E* rendered the plants resistant to tobacco etch virus (TEV), turnip mosaic virus (TuMV), and lettuce mosaic virus (LMV) (Duprat et al. 2002; Lellis et al. 2002). Further studies revealed similar mechanisms in crop species, identifying mutations in *eIF4E* or *eIFiso4E* as the genetic basis for potyvirus resistance in various cultivars, such as *pvr2* in pepper (Ruffel et al. 2002), *pot1* in tomato (Ruffel et al. 2005), *mo1* in lettuce (Nicaise et al. 2003), *sbm1* in pea (Gao et al. 2004), *bc-3* in common bean (Nakahara et al. 2010), *nsv* in melon (Nieto et al. 2006), and *rym4* in barley (Kanyuka et al. 2005). Recessive resistance conferred by these naturally occurring alleles, or by loss-of-function *eIF4E*/*eIFiso4E* mutants generated through mutagenesis, has been successfully used in breeding programs to control potyviral diseases (Robaglia and Caranta 2006; Wang and Krishnaswamy 2012; Bastet et al. 2017).

In plants, members of the small eIF4E family exhibit functional redundancy due to gene duplication events, which may provide multiple potential susceptibility factors for viral infections (Dinkova et al. 2016). Within the family *Potyviridae*, some potyviruses rely exclusively on specific eIF4E isoforms, while others utilize both eIF4E and eIFiso4E for infection (Wang and Krishnaswamy 2012; Zlobin and Taranov 2023). Consequently, mutations in a single *eIF4E* family gene often result in a limited resistance spectrum (Gauffier et al. 2016; Bastet et al. 2017; Moury et al. 2020). Moreover, potyviruses have a remarkable ability to adapt to resistance mechanisms by evolving mutations, typically in the VPg protein. These mutations enable the resistance-breaking (RB) variants to re-establish compatible interactions with previously resistant *eIF4E* alleles or alternative *eIF4E* isoforms in the host (Borgstrom and Johansen 2001; Moury et al. 2004, 2014; Ayme et al. 2006; Bruun-Rasmussen et al. 2007; Perez et al. 2012; Lebaron et al. 2016). Thus, understanding the dynamics of VPg-eIF4E interactions and the molecular basis of resistance breakdown is crucial for devising sustainable resistance mechanisms (Wang and Krishnaswamy 2012; Yang et al. 2021; Zlobin and Taranov 2023).

In tobacco (*Nicotiana tabacum* L.), the recessive *va* locus has long been recognized for its ability to confer resistance or tolerance to potyviruses such as potato virus Y (PVY), tobacco vein mottling virus (TVMV), and TEV. The *va* resistance locus originates from the Virgin A Mutant (VAM) tobacco line, generated via UV-induced mutagenesis, which caused a large ~ 1 Mb deletion on chromosome 21 (Koelle 1961). This deletion resulted in the loss of the *eIF4E1* gene copy inherited from the *N. sylvestris* ancestor (*eIF4E1-S*), which was identified as the key genetic determinant for PVY resistance (Julio et al. 2014; Dluge et al. 2018). The *va* locus has been extensively deployed in commercial tobacco cultivars, where it has successfully controlled PVY outbreaks for decades. However, the emergence of PVY RB isolates in fields deployed with *va*-resistant tobacco has led to cases of resistance breakdown (Masuta et al. 1999; Lacroix et al. 2011; Acosta-Leal and Xiong 2013; Moury et al. 2014). A recent study identified that inactivation of *eIFiso4E-T,* an isoform inherited from the *N. tomentosiformis* ancestor, reduced but did not eliminate susceptibility to PVY RB in the *Va* genetic background (Takakura et al. 2018). Furthermore, quadruple mutants inactivating *eIF4E1-S*, *eIF4E1-T*, *eIF4E2-S*, and *eIF4E2-T* were reported to confer long-lasting PVY resistance in tobacco (Le et al. 2022). Thus, the molecular basis underlying the *va* resistance breakdown remains obscure.

Here, we first report on the survey of potyviral diseases in *va* tobacco fields in Yunnan, China, revealing the emergence of PVY RB isolates and the prevalence of two other potyviruses, tobacco vein banding mosaic virus (TVBMV) and chilli veinal mottle virus (ChiVMV). Using protein interaction assays and genetic mutants of the *eIF4E* family members, we identified both eIF4E1-S and eIFiso4E-T as key susceptibility factors for PVY RB. In tobacco with the *va* genotype, inactivation of *eIFiso4E-T* provided durable resistance to PVY, while knocking out *eIFiso4E-S* conferred resistance or tolerance to TVBMV and ChiVMV. Moreover, the triple mutant (*va eifiso4e-t eifiso4e-s*) displayed effective resistance to all three potyviruses without significant developmental defects. This study underscores the feasibility of stacking *eIF4E*/*eIFiso4E* mutations to engineer broad-spectrum and durable resistance to potyviruses in tobacco.

## Results

### Field survey of potyviruses in tobacco *va* and *Va* varieties in Yunnan province

Commercial tobacco (*N. tabacum*) cultivars carrying the introgressed *va* locus, which confers recessive resistance to PVY, have been widely cultivated for over 15 years in Yunnan province, the leading tobacco-growing region in China. To evaluate the effectiveness of *va*-mediated resistance against PVY and to assess the prevalence of potyviral diseases, a field survey was conducted from 2016 to 2019 across 13 major tobacco-growing counties in Yunnan. In total, 497 tobacco leaf samples showing potyvirus-like symptoms were collected. Among these, 226 samples were from PVY-resistant *va* genotypes (NC102, Yunyan301, and Yunyan121), while 271 were from PVY-susceptible *Va* genotypes (Yunyan116, Yunyan87 (Y87), and K326). These samples were subjected to enzyme-linked immunosorbent assay (ELISA) assay using antibodies specific to the three prevalent potyviruses in the region: PVY, TVBMV, and ChiVMV.

**Fig. 1:**
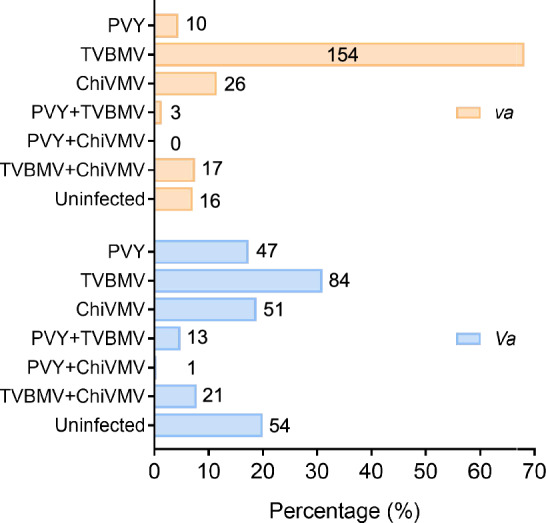
Detection of PVY, TVBMV, and ChiVMV in field-collected tobacco samples by ELISA. A total of 226 leaf samples from PVY-resistant (*va*) and 271 samples from PVY-susceptible (*Va*) tobacco genotypes were collected from 13 major tobacco-growing counties in Yunnan province, China, between 2016 and 2019. The number of samples infected singly or doubly with potyviruses or uninfected is indicated on each column

Overall, 85.9% (427 out of 497) of samples tested positive for at least one potyvirus. Notably, the *va*-containing resistant cultivars showed significantly reduced PVY incidence and yield loss in high-risk areas. PVY single or mixed infections were detected in only 5.8% (13/226) of *va* samples, compared to 22.5% (61/271) in *Va* genotypes (Fig. 1). However, TVBMV emerged as the predominant potyvirus in *va* cultivars, accounting for 68.1% (154/226) of samples with single-infection cases. Additionally, mixed infections of TVBMV with ChiVMV or PVY were found in 7.5% (17/226) and 1.3% (3/226) of *va* samples, respectively.

These findings suggest that while *va*-mediated resistance remains effective against PVY, the emergence of potential PVY RB strains and the prevalence of other related potyviruses, such as TVBMV and ChiVMV, have led to continued potyvirus epidemics in Yunnan Province. This underscores the urgent need to develop more durable, broad-spectrum resistance to ensure long-term control of potyviruses in tobacco.

### K105 mutation in the VPg cistron of PVY determines resistance breaking in *va* tobacco

To investigate the mechanism underlying *va* resistance breakdown, we mechanically inoculated PVY-ZT5, an isolate collected from a *Va* tobacco plant in Yunnan and belonging to the PVY^N^ strain (GenBank Accession no. MF960848) (Sun et al. 2017), onto TN86 (*va* genotype) and Hongda (*Va* genotype) tobacco plants. While systemic infection was uniformly observed in Hongda plants, starting approximately 7 days post-inoculation (dpi), the inoculated TN86 plants displayed sporadic systemic infections at later stages of infection. Previous studies have shown that spontaneous mutations in the VPg protein of various potyviruses can overcome *eIF4E*-mediated resistance in several plant species (Nicolas et al. 1997; Masuta et al. 1999; Moury et al. 2004; Kang et al. 2005; Ayme et al. 2006; Charron et al. 2008; Janzac et al. 2014; Takakura et al. 2018). Therefore, we cloned and sequenced the VPg coding region amplified from PVY-infected TN86 and Hongda plants. Sequence alignment of 23 TN86-derived VPg clones and 10 Hongda clones revealed the presence of a lysine-to-glutamine substitution at position 115 (K105Q) in all TN86 clones. Additionally, an isoleucine to valine substitution, at position 139 (I139V), was identified in all TN86 clones but also occurred in 3 out of 10 Hongda clones (Fig. S1).

**Fig. 2:**
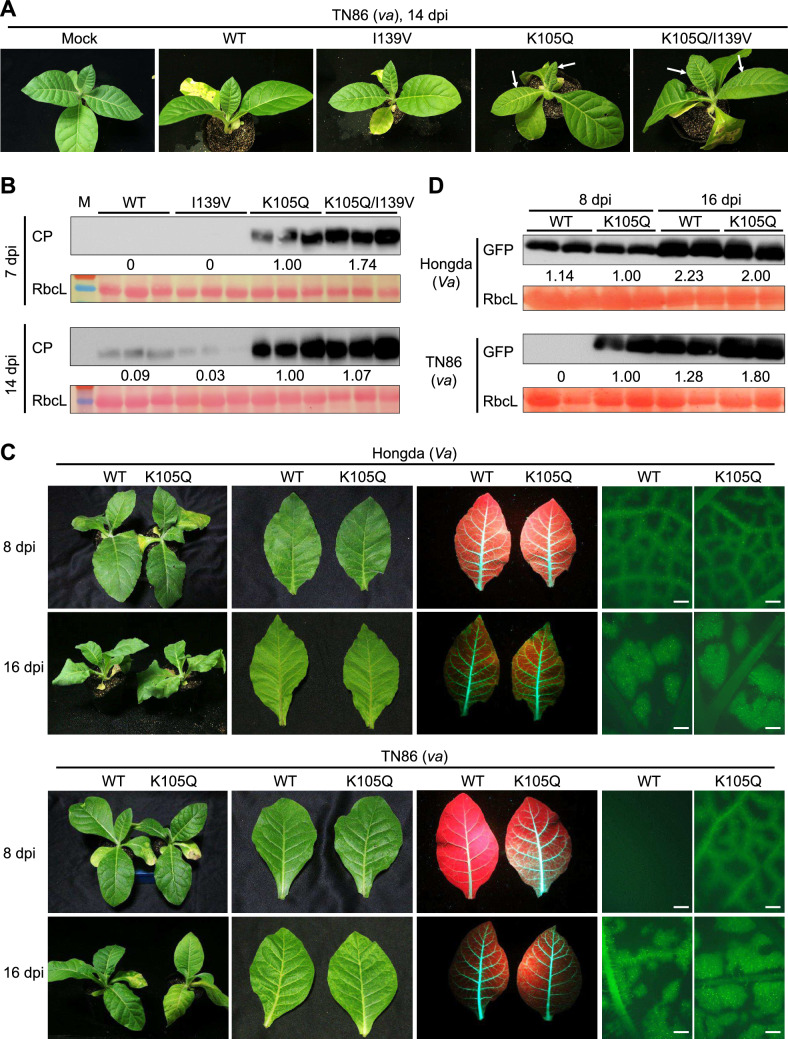
Identification of VPg mutations required for breaking the *va*-mediated PVY resistance. **A, B** Infection of TN86 tobacco carrying the *va* resistance locus via agroinoculation of PVY wild-type (WT) clone or its derived mutants I139V, K105Q, or K105Q/I139V. **A** Photographs of inoculated plants at 14-days post inoculation (dpi). For each virus, 16 plant were inoculated, and representative images are shown. **B** Immunoblots showing PVY coat protein (CP) accumulation in upper young leaf tissues at 7 and 14 dpi. Rubisco large subunit (RbcL), stained with Ponceau S, serves as protein loading controls. **C, D** Infection of Hongda and TN86 plant via agroinoculation with wild type PVY-GFP or K105Q mutant clones. **C** Photographs of inoculated plants and upper leaves at 8 and 16 dpi, under normal light or UV radiation, or viewed via fluorescent microscope. Scale bars, 500 μm. **D** Immunoblots showing GFP expression in upper non-inoculated leaves. The protein levels in (**B**) and (**D**) were quantified using image J software. Relative protein accumulation levels, normalized against the values of the K105Q samples, are indicated below each panel. Experiments shown in (**C**) and (**D**) were repeated three times, each with *n* = 12 biological replicates (individual plants) per group, and similar results were obtained

To evaluate the contribution of these VPg mutations to *va* resistance breakdown, we introduced the K105Q, I139V, and K105Q/I139V double mutations into the PVY-ZT5 infectious clone. The resultant mutant viruses, along with the wild type (WT) PVY-ZT5, were agroinoculated onto TN86 plants. Systemic symptoms appeared in plants inoculated with the PVY K105Q or K105Q/I139V mutants as early as 7 dpi, and all 16 inoculated plants for each virus developed severe symptoms by 14 dpi. In contrast, plants inoculated with PVY WT or the I139V mutant only started to exhibit systemic symptoms at 14 dpi (Fig. 2A). Western blot analyses confirmed that PVY WT and I139V accumulated little to no coat protein (CP) at 7 dpi and low levels of CP even at 14 dpi. Conversely, the PVY K105Q and K105Q/I139V mutants accumulated high levels of CP at 7 dpi and 14 dpi (Fig. 2B).

To visualize the infection process, we introduced the K105Q mutation into the PVY-GFP clone carrying a fluorescent reporter gene (Sun et al. 2017). Symptom observation, fluorescence microscopy, and protein blot analyses indicated that PVY-GFP and its K105Q mutant exhibited comparable virulence in Hongda plants, indicating no differences in their ability to infect this susceptible genotype. However, in TN86 plants carrying the *va* resistance locus, the parental PVY-GFP strain showed a ~ 7-day delay in systemic infection, compared to the PVY-GFP K105Q mutant (Fig. 2C and D). Leaf tissues from five TN86 plants infected with PVY-GFP were pooled and the cDNA sequence of VPg determined, which revealed mutations at K105 in all clones, including glutamic acid (K105E, 7/10), glutamine (K105Q, 1/10), threonine (K105T, 1/10), or methionine (K105M, 1/10), alongside with the I131V mutation detected in 2/10 clones (Table 1). No mutations were detected in the VPg sequences from PVY-GFP progeny propagated in Hongda plants or from PVY-GFP K105Q progeny in either host genotype (Table 1). Our results demonstrate that PVY RB variants spontaneously arise in *va* tobacco and that mutations at K105 in VPg are critical for overcoming *va*-mediated resistance. This is consistent with prior studies (Masuta et al. 1999; Janzac et al. 2014; Michel et al. 2019), which implicated K105 mutations in PVY VPg in breaking *va* resistance. The I139V mutation, on the other hand, appears to play a negligible or minor role in this process. Hence, we designate the K105Q mutant as the PVY RB strain hereafter.

**Table 1:** Amino acid changes identified in the PVY VPg protein after infection of susceptible and resistant tobacco accessions

Infectious clone	Host genotype	No. 105 aa	No. 139 aa
PVY-GFP WT	Hongda (*Va*)	K (10/10)	I (10/10)
	TN86 (*va*)	E (7/10), Q (1/10) T (1/10), M (1/10)	I (8/10), V (2/10)
PVY-GFP K105Q	Hongda (*Va*)	Q (5/5)	I (5/5)
	TN86 (*va*)	Q (5/5)	I (5/5)

### Screening of eIF4E homologs interacting with the VPg K105Q mutant protein

**Fig. 3:**
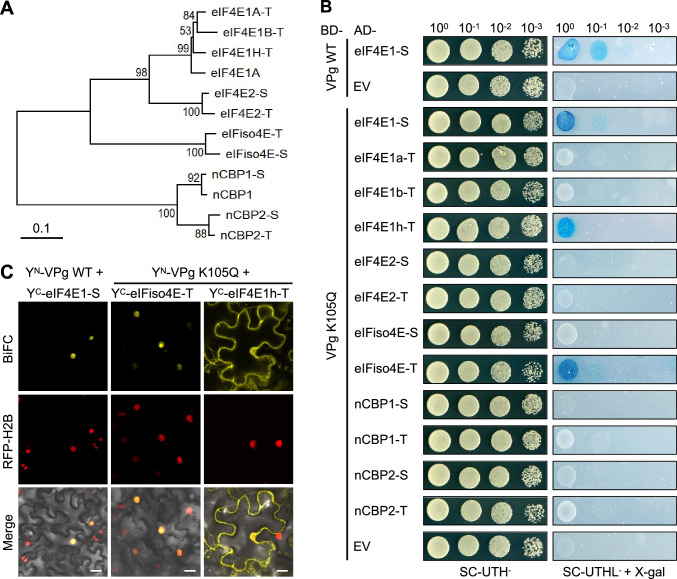
Screening of eIF4E family members interacting with VPg K105Q. **A** Phylogenetic analysis of the eIF4E family members in *Nicotiana tabacum*. The suffix “-T” and “-S” denote derivation from the parental genome of *N. tomentosiformis* and *N. sylvestris*, respectively. **B** Yeast two-hybrid analysis of the interaction between eIF4E members and VPg WT and K105Q mutant. Yeast strains, transformed with bait and prey constructs, were serially diluted and spotted on non-selective (SC-UTH^−^) and selective media (SC-UTHL^−^) with X-gal. **C** BiFC assay confirming of VPg-eIF4E interactions. Leaves of *N. benthamiana* RFP-H2B plants were agroinfiltrated to co-express YFP N terminus (Y^N^) fused to eIF4E members and YFP C terminus (Y^C^) fused to VPg. Confocal micrographs were captured at 48 h post-infiltration (hpi). Scale bars, 20 μm

Previous studies have shown that the breakdown of *eIF4E*-mediated resistance involves the restoration of interactions between VPg mutants and alternative eIF4E isoforms (Takakura et al. 2018; Yang et al. 2021; Zlobin and Taranov 2023). A BLAST search identified 12 *eIF4E* family members in the *N. tabacum* genome. Phylogenetic analysis classified these proteins into four clades: eIF4E1, eIF4E2, eIFiso4E, and novel cap-binding protein (nCBP) (Julio et al. 2014; Fig. 3A). Using previously defined nomenclature (Takakura et al. 2018; Le et al. 2022), the eIF4E1 clade was found to consist of four closely related members: eIF4E1-S (Va) encoded in the S subgenome derived from the ancestral *N. sylvestris* and three paralogs (eIF4E1a-T, eIF4E1b-T, and eIF4E1h-T) encoded by the T subgenome originating from *N. tomentosiformis* through a gene triplication event (Michel et al. 2019). Additionally, both S and T subgenomes encode one eIF4E2 (eIF4E2-S and eIF4E2-T), one eIFiso4E (eIFiso4E-S and eIFiso4E-T), and two nCBPs (nCBP1-S, nCBP1-T, nCBP2-S, and nCBP2-T) members.

We hypothesized that PVY RB might recruited one or more specific eIF4E isoforms to establish productive infection in *va* tobacco, which lack the PVY-susceptible *eIF4E1-S* (Julio et al. 2014). Supporting this hypothesis, Y2H assays revealed specific interactions between VPg K105Q and eIF4E1-S, eIF4E1h-T, and eIFiso4E-T, as well as the expected interaction between VPg WT and eIF4E1-S (Fig. 3B).

BiFC assays further validated these interactions. As shown Fig. 3C, strong fluorescence was observed in *N. benthamiana* epidermal cells co-expressing VPg WT with eIF4E1-S and VPg K105Q with eIFiso4E-T. Consistent with prior findings (Beauchemin et al. 2007), the fluorescent signals were targeted exclusively to the nucleus. Interestingly, BiFC signals of VPg K105Q and eIF4E1h-T interaction were predominantly localized to the cytoplasm (Fig. 3C). It has been previously proposed that eIF4E1h-T may function as a decoy to interact with PVY VPg in a non-productive manner, thereby increasing the durability of *va*-mediated resistance (Michel et al. 2019). Therefore, we hypothesize that eIFiso4E-T could act as a susceptibility factor exploited by PVY RB in *va* tobacco.

### CRISPR/Cas9-mediated mutation of *eIFiso4E-T* and *eIFiso4E-S*

**Fig. 4:**
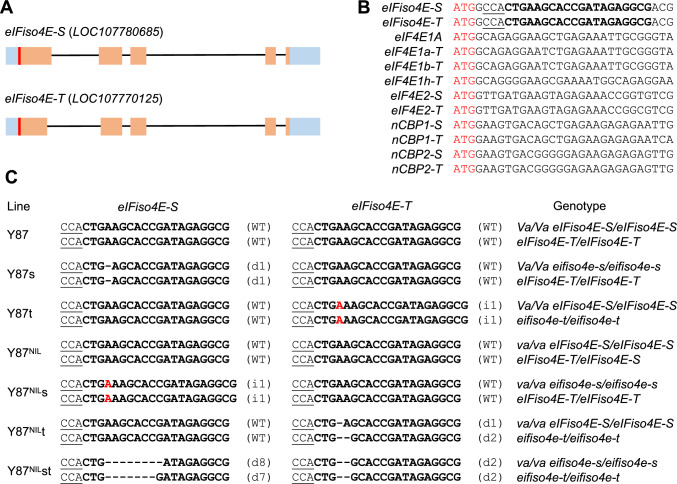
CRISPR/Cas genome editing of *eIFiso4E-T* and *eIFiso4E-S* in tobacco genotypes Y87 (*Va*/*Va*) and Y87^NIL^ (*va*/*va*). **A** Schematic diagram of *eIFiso4E-T* and *eIFiso4E-S* gene structure. Light blue and orange boxes represent non-coding and coding exons, line indicate intron, and red bars denote target sites. **B** Alignment of the target site sequences in the first exon. Start codons ATG are highlighted in red, PAM sites underlined and protospacer sequences in bold. **C** Genotypes of tobacco knockout lines. Dashes represent nucleotide deletions, and letter labeled in red indicated nucleotide insertions. d# and i# denote the number of base deletion and insertion, respectively. Uppercase letters in the “genotype” column indicate wild type genes, while lowercase letters indicate mutated alleles

Due to the high sequence similarity (92% nucleotide sequence identity) between *eIFiso4E-T* and *eIFiso4E-S* (Fig. 4A and S2), designing a specific single guide RNA (sgRNA) to exclusively target *eIFiso4E-T* pose a challenge. We hypothesized that a single sgRNA targeting both genes might generate *eifso4e-t* and *eifso4e-s* single mutants, given that gene editing might not be efficient. To this end, a 20-nucleotide sgRNA spacer sequence located downstream of the ATG start codon, which specifically targets both *eIFiso4E-T* and *eIFiso4E-S*, was selected (Fig. 4B).

We transformed the Y87 (*Va* genotype) and a near-isogenic line (NIL) of Y87 (Y87^NIL^) containing the introgressed *va* locus with the Cas9/sgRNA binary construct, yielding 22 transgenic T_0_ lines for Y87 and 55 lines for Y87^NIL^. Genotyping by sequencing of target site amplicons revealed small nucleotide deletions or insertions in *eIFiso4E-S* or *eIFiso4E-T* in some transgenic lines (Fig. 4C). We selected lines harboring homozygous or bi-allelic frameshift mutations in *eIFiso4E-S* and/or *eIFiso4E-T*, resulting in the following genotypes: Y87s (*eifso4e-s*) and Y87t (*eifso4e-t*) in Y87 (*Va*) background, and Y87^NIL^s (*eifso4e-s*), Y87^NIL^t (*eifso4e-t*), and Y87^NIL^st (*eifso4e-s eifso4e-t*) in Y87^NIL^ (*va*) background (Fig. 4C). These T_0_ lines were self-pollinated, and transgene-free T_1_ progeny seedlings were used for subsequent analysis.

### Knocking out *eIFiso4E-T* in the *va* background confers durable resistance to PVY

**Fig. 5:**
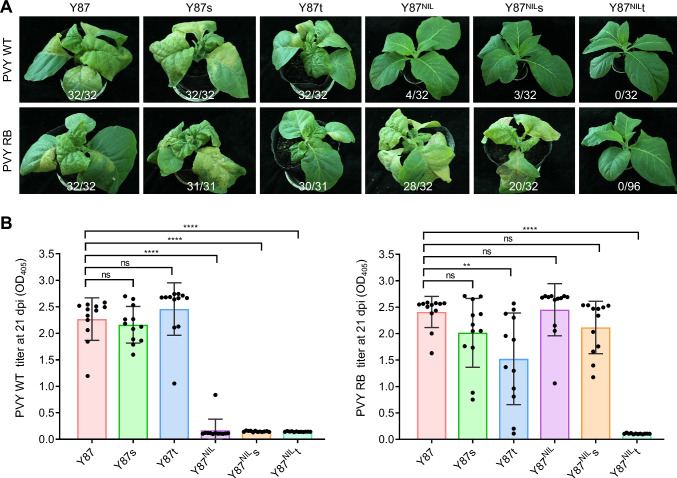
Analysis of resistance in *eIFiso4E*-edited tobacco lines to PVY infection. **A** Symptom development in tobacco lines infected with PVY WT or RB strains. Plants were mechanically inoculated with PVY leaf sap and photographed at 14 dpi. Numbers on each panel denote the number of symptomatic plants out of the total number of inoculated plants. Data are representative of four independent experiments (*n* = 8 or 24 plants per group, per experiment). **B** Bar chart showing the titers of PVY WT (left) and PVY RB (right) in inoculated tobacco plants, determined by ELISA assays. Twelve plants from each line, irrespective of symptom presence, were analyzed at 21 dpi. Four leaf discs per plants from upper leaves at similar developmental stages were pooled and analyzed. Data are presented as individual data points with mean ± SD (*n* = 12). Statistical significance was determined using two-tailed Student's *t*-test (**, *P* < 0.01; ****, *P* < 0.0001; ns, *P* > 0.05 not significant)

To assess the impact of *eIFiso4E-T* and eIFiso4E-S knockouts on PVY resistance, T_1_ progeny of Y87, Y87s, Y87t, Y87^NIL^, Y87^NIL^s, and Y87^NIL^t genotypes were rub inoculated with PVY WT leaf saps. Three lines (Y87, Y87s, Y87t) with the *Va* genotype, irrespectively of the presence of *eifso4e-s* or *eifso4e-t* knockouts, exhibited complete susceptibility to PVY WT. In contrast, all three Y87^NIL^-derived lines showed no or reduced susceptibility to PVY WT, underscoring the critical role the *va* locus in conferring resistance. Specifically, 4 out of 32 Y87^NIL^ plants and 3 out of 32 Y87^NIL^s plants developed systemic PVY infection at 14 dpi. More remarkably, all 32 inoculated Y87^NIL^t plants remained symptom-free throughout the entire growth stages (Fig. 5A, upper panels), highlighting the essential role of *eifso4e-t* in maintaining durable *va*-mediated resistance. ELISA analysis of virus titers in upper leaves of inoculated tobacco at 21 dpi confirmed minimal or no PVY accumulation in *va* genotype tobacco lines, while high viral titers were detected in the three *Va* lines (Fig. 5B).

To further evaluate resistance, we mechanically inoculated these lines with PVY RB. Although all three Y87-derived lines showed susceptibility, virus symptoms in infected Y87t plants were noticeably milder compared to those in Y87 or Y87s (Fig. 5A, lower panels). Among the three lines with the *va* genotype, 88% (28/32) of Y87^NIL^ displayed typical infection symptoms, while the infection rate dropped to 63% (20/32) in Y87^NIL^s plants. Strikingly, none of the 96 inoculated Y87^NIL^st plants developed PVY symptoms throughout the duration of the experiment (Fig. 5A, lower panels). ELISA assays conducted at 21 dpi confirmed undetectable PVY titers in Y87^NIL^st plants. Additionally, modest but statistically significant reductions in PVY titers were observed in Y87t plants, whereas the other tobacco genotypes displayed high virus titers (Fig. 5B). These results demonstrate that *eIFiso4E-T* serve as critical susceptible gene for PVY RB and that its knockout in the *va* genetic background confers effective resistance to PVY WT and RB strains. In contrast, the knockout of *eIFiso4E-S* had negligible effects on PVY infection.

### Knocking out *eIFiso4E-S* in the *va* background confers broad resistance to TVBMV and ChiVMV

**Fig. 6:**
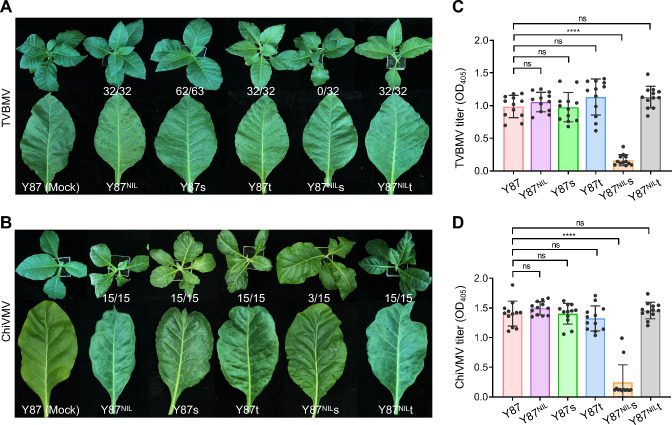
Analysis of resistance in *eIFiso4E*-edited tobacco lines to TVBMV and ChiVMV infections. **A, B** Symptom development in tobacco lines infected with (**A**) TVBMV and (**B**) ChiVMV. Photographs of mechanically inoculated plants and upper, uninoculated leaves were taken at 14 dpi. Numbers on each panel denote the number of symptomatic plants out of the total number of inoculated plants. Mock, mock-inoculated plants. Data in (**A**) are representative of four independent experiments (*n* = 8 or 16 plants per group per experiment) and Data in (**B**) are representative of three independent experiments (*n* = 5 plants per group per experiment). **C, D** Bar charts displaying (**C**) TVBMV and (**D**) ChiVMV titers in inoculated tobacco plants, determined by ELISA assays. For each line, twelve plants with or without viral symptoms, were analyzed at 21 dpi. Data are presented as individual data points with mean ± SD (*n* = 12). Statistical comparisons were performed using two-tailed Student's *t*-test (****, *P* < 0.0001; ns, *P* > 0.05 not significant)

We also assessed the susceptibility of the aforementioned *eIF4E* mutant lines to TVBMV and ChiVMV, two emerging potyviruses in Yunnan, China. Following rub inoculation with TVBMV saps, nearly all inoculated plants of Y87, Y87s, Y87t, Y87^NIL^, and Y87^NIL^t exhibited systemic symptoms at 14 dpi. Remarkably, Y87^NIL^s showed no viral symptoms in any of the 32 inoculated plants (Fig. 6A). Similarly, Y87^NIL^s displayed resistance to ChiVMV upon sap inoculation. Only 20% (3/15) of inoculated plants in this line developed systemic infections, compared to 100% infection rates (15/15) observed in the other five genotypes (Fig. 6B). ELISA assays quantifying virus titers in upper leaf tissues further supported these observations, confirming robust resistance of Y87^NIL^s to TVBMV and moderate resistance to ChiVMV (Fig. 6C and D).

These results demonstrate that knocking out either *eIF4E-S* or *eIFiso4E-S* alone is insufficient to confer resistance to TVBMV or ChiVMV. However, the double knockout *eif4e-s eifiso4e-s* results in loss of or reduced susceptibility to both viruses. Thus, these data suggest that TVBMV and ChiVMV could utilize both eIF4E-S and eIFiso4E-S for productive infections.

### Triple mutant *va eifiso4e-s eifiso4e-t* conferred durable and broad-spectrum resistance to potyviruses without obvious developmental defects

Given that two *eIFiso4E* genes (*eIFiso4E-S* and *eIFiso4E-T*) are differentially required for infections by PVY and TVBMV/ChiVMV, we hypothesized that their simultaneous knockouts in the *va* genetic background would confer broad-spectrum resistance to these potyviruses. To test this, we rub inoculated Y87^NIL^st and Y87^NIL^ plants with PVY, TVBMV and ChiVMV separately and monitored symptom development.

**Fig. 7:**
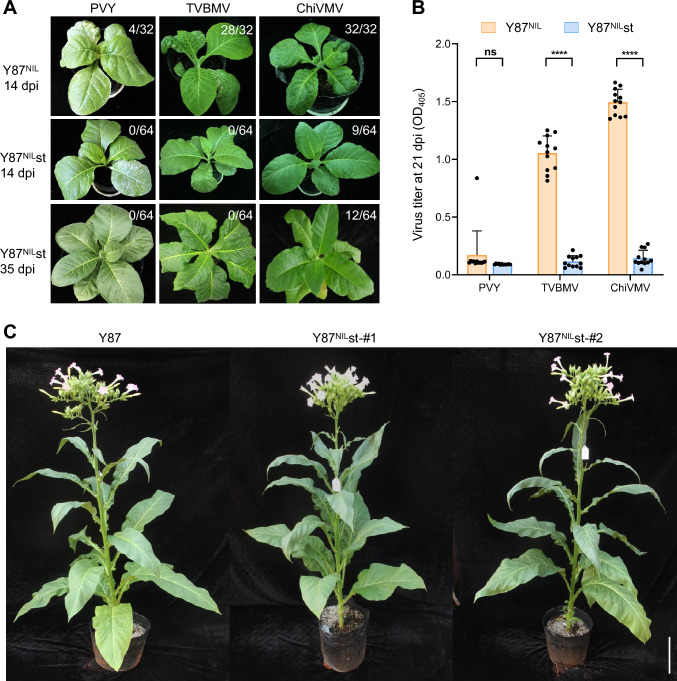
Resistance of the *va eifiso4e-t eifiso4e-s* triple mutant lines to three potyviruses. **A** Symptom development in Y87^NIL^ (*va*) and Y87^NIL^st (*va eifiso4e-t eifiso4e-s*) following mechanical inoculation with PVY, TVBMV, and ChiVMV. Numbers on each panel denote the number of symptomatic plants out of the total number of inoculated plants. Data are representative of four independent experiments (*n* = 8 or 16 plants per group per experiment). **B** Quantification of virus titers in inoculated plants at 21 dpi by ELISA. Data are presented as individual data points with mean ± SD (*n* = 12). Statistical significance was evaluated using two-tailed Student's *t*-test (****, *P* < 0.0001; ns, *P* > 0.05 not significant). **C** Comparison of the growth phenotypes of Y87 and Y87^NIL^st plants. Flowering plants at 150 days post germination were photographed. Scale bar, 10 cm

In Y87^NIL^ plants containing the *va* locus alone, we observed partial resistance to PVY and full susceptibility to ChiVMV and TVBMV. Specifically, 12.5% (4/32), 87.5% (28/32), and 100% (32/32) of inoculated plants, respectively, developed systemic symptoms, including vein necrosis and necrotic spots, by 14 dpi (Fig. 7A). By contrast, none of the 64 inoculated Y87^NIL^st plants displayed any viral symptoms when infected with PVY or TVBMV at 14 dpi and even 35 dpi, indicating effective and durable resistance to these viruses. Additionally, Y87^NIL^st showed reduced susceptibility to ChiVMV, with only 14.1% (9/64) of the plants showing mild symptoms at 14 dpi and 18.75% (12/64) at 35 dpi (Fig. 7A). ELISA assays confirmed these observations by showing low or background levels of virus titers in Y87^NIL^st plants inoculated with TVBMV and ChiVMV, as well as in both genotypes inoculated with PVY (Fig. 7B).

Under greenhouse growth conditions, Y87^NIL^st plants exhibited normal vegetative development and fertility. Preliminary growth evaluation revealed that plant height, flowering time, and leaves number of Y87^NIL^st plants were comparable to the parental Y87 line (Fig. 7C). These results indicate that pyramiding *eIF4E-S* and *eIFiso4Es* mutations in tobacco provide a feasible strategy to achieve durable and broad-spectrum resistance to potyviruses without causing major growth penalty.

## Discussion

Our field survey revealed that while the deployment of tobacco cultivars carrying the introgressed *va* resistance locus reduced PVY incidence in Yunnan, the emergence of potential PVY RB isolates, along with TVBMV and ChiVMV, presents significant challenges for sustainable potyvirus control. Both TVBMV and ChiVMV have recently emerged in southwestern and southern China, causing severe yield losses in solanaceous crops, including tobacco, pepper, potato, and tomato (Ding et al. 2011; Zhang et al. 2011). Currently, no resistance genes have been identified for either TVBMV or ChiVMV, emphasizing the urgent need for innovative resistance strategies. CRISPR/Cas genome editing technology offers a powerful tool for engineering disease resistance by precisely modifying host susceptibility genes, which has the potential to confer broader and more durable resistance than the dominant, race-specific resistance genes (van Schie and Takken 2014; Dong and Fang 2024). To maximize its effectiveness, it is crucial to identify susceptibility genes exploited by different potyviruses and elucidate the mechanisms of resistance- breaking.

Our findings demonstrate that PVY RB variants spontaneously emerged in *va* tobacco following sap or agroinoculation, although resistance breakdown occurred more efficiently during agroinoculation, likely due to the higher amount of viral RNA introduced into cells. Notably, in contrast to the exclusive K105Q mutation detected in progeny PVY-ZT5 genomes after sap inoculation of *va* tobacco, agroinoculation with the cDNA clone resulted in RB variants predominantly carrying the K105E mutation (7/10), along with low frequencies of K-to-Q, M, or T mutations (1/10 each). The different VPg mutation spectra is likely due to differences in inoculum sources. Unlike the infectious cDNA clone with a single defined sequence, virus populations in leaf sap exist as quasispecies. The PVY-ZT5 sap was derived from a field sample collected from Yunnan province where *va* and *Va* tobacco genotypes were co-cultivated and then maintained on susceptible (*Va*) tobacco. It is possible that the K105Q RB variant was already present at low abundance in the PVY-ZT5 sap inoculum and subsequently became dominant under the selection pressure imposed by *va* resistance. Indeed, Janzac et al (2014) demonstrated that PVY RB variants carrying adaptive mutations at VPg position 105 could revert to wild type when propagated on susceptible tobacco lines, as evidenced by the detection of a 105K/105E mixture in individual plants. Nevertheless, all these mutation types have been previously observed in RB population of various PVY strains, with K105E being the most prevalent (Masuta et al. 1999; Janzac et al. 2014; Takakura et al. 2018; Michel et al. 2019). As observed in our study for K105Q, each of the K105E, K105T, or K105M mutations has been shown to enable PVY infection in *va* tobacco (Janzac et al. 2014), suggesting that these variants employ similar resistance-breaking mechanisms.

Using the VPg K105Q mutant protein as bait, we observed specific interactions with three eIF4E proteins: eIF4E1-S, eIF4E1h-T, and eIFiso4E-T. The interaction with eIF4E1-S explains the sustained virulence of PVY RB in *Va* genotype tobacco, which exhibited symptom onset rates comparable to the parental strain. The interaction between VPg K105Q and eIFiso4E-T aligns with prior findings, where eIFiso4E-T was shown to interact with VPg K105E and S101G/V108I mutants of PVY RB isolates (Takakura et al. 2018). Utilizing CRISPR/Cas9-generated mutant lines, we demonstrated that inactivating *eIFiso4E-T* in the *va* genetic background not only conferred durable resistance to PVY WT but also blocked infection by PVY RB variants. These results conclusively establish both eIF4E1-S and eIFiso4E-T as essential susceptibility factors for PVY RB.

The interaction of VPg K105Q with eIF4E1h-T is noteworthy, as eIF4E1h-T has been proposed to act as a decoy for VPg, thereby enhancing the durability of resistance conferred by *eIF4E1-S* deletion (Michel et al. 2019). The expression of *eIF4E1h-T*, previously referred to as *eIF4E-2* or *T021658*, was highly induced in resistant VAM accessions lacking *eIF4E1-S* (Julio et al. 2014). Furthermore, the durability of *va* resistance in tobacco correlates positively with *eIF4E1h-T* expression levels, which vary depending on the type of mutation at the *va* locus, such as deletions of various sizes, frameshift or nonsense mutations. Notably, while VPg is known to target the nucleus and nucleolus (Restrepo et al. 1990; Beauchemin et al. 2007), we found that VPg K105Q interacted with eIF4E1h-T predominantly in the cytoplasm, whereas its interaction with eIF4E1-S localized to the nucleus. While the precise mode(s) of action of eIF4E in potyvirus infection has not been fully elucidated, it has been proposed that nuclear VPg-eIF4E interaction is involved in processes critical for viral systemic spread, such as suppression of eIF4E-mediated nuclear mRNA export or host defense mechanisms (Wang and Krishnaswamy 2012). Our data suggests that the upregulated eIF4E1h-T in *va* tobacco may sequester VPg in the cytoplasm and prevent it from interacting with eIFiso4E-T in the nucleus, thereby limiting the appearance of RB variants to confer durable resistance.

Similar to PVY RB, our data revealed that TVBMV and ChiVMV exploit two eIF4E isoforms, specifically eIF4E1-S and eIFiso4E-S, for productive infection. Single knockout mutations in either *eIF4E1-S* or *eIFiso4E-S* did not confer resistance to these viruses. These findings are consistent with earlier research showing that ChiVMV VPg interacts with both eIF4E and eIFiso4E homologs in pepper, and mutations in both genes are required for effective resistance (Hwang et al. 2009).

Inactivation of both *eIFiso4E-T* and *eIFiso4E-S* in the *va* background yielded the triple mutant (*va eifiso4e-t eifiso4e-s*), which displayed robust resistance to all three potyviruses, i.e., PVY, TVBMV, and ChiVMV. These findings highlight the potential of targeting specific eIF4E isoform combinations to enhance both the durability and spectrum of resistance. While combining multiple eIF4E knockouts has often resulted in developmental abnormalities such as dwarfism, delayed bolting, and male sterility in species like *Arabidopsis*, tomato, and melon (Bastet et al. 2017), the *va eifiso4e-t eifiso4e-s* triple mutant displayed no discernible growth or fertility defects under standard greenhouse conditions. This is likely due to the compensatory roles of the remaining five eIF4E copies in allotetraploid tobacco. Nonetheless, further studies are required to evaluate the performance of these mutants under diverse field conditions to confirm their agronomic viability.

In conclusion, while K105 mutations in VPg are known to break va resistance (Masuta et al. 1999; Janzac et al. 2014; Michel et al. 2019), our study advances this knowledge by elucidating novel susceptibility factors and engineering unprecedented broad-spectrum resistance. We identified eIFiso4E-T as a critical host factor enabling PVY RB infection in *va* tobacco, alongside confirming *eIFiso4E-S* as a key susceptibility factor for TVBMV and ChiVMV infections. By stacking mutations in these genes, we generated the *va eifiso4e-t eifiso4e-s* triple mutant, which confers robust resistance to three economically significant potyviruses—PVY, TVBMV, and ChiVMV—without compromising plant growth or fertility under greenhouse conditions. Furthermore, our findings are grounded in field data from Yunnan Province, where the emergence of PVY RB isolates and the prevalence of TVBMV/ChiVMV underscore the urgency of deploying multi-layered resistance strategies. This study provides a blueprint for engineering durable, broad-spectrum resistance in allotetraploid crops by exploiting functional redundancies within the eIF4E family, offering a practical solution to mitigate viral epidemics in agricultural systems.

## Materials and methods

### Plant materials and infectious cDNA clones

The PVY-susceptible tobacco (*N. tabacum*) genotypes (*Va/Va*), Hongda and Yunyan 87 (Y87), and the resistant genotypes (*va/va*), TN86 and Y87^NIL^ carrying the *va* locus, were used for potyvirus inoculation. Y87^NIL^ line was developed through recurrent backcrossing and selfing (BC_5_F_4_) in the genetic background of Y87.

The infectious cDNA clone of the PVY ZT-5 isolate (Genbank Accession no. MF960848) belonging to the N strain group, and a derivative containing a *green fluorescent protein* (*GFP*) gene inserted between the NIb and CP cistrons GFP (PVY-GFP), were described previously (Sun et al. 2017). The VPg K105Q, I139V, and K105Q/I139V double mutations were introduced to the PVY and PVY-GFP infectious clones by site-directed mutagenesis to generate the PVY RB clones. The PVY RB strain was maintained on TN86 plants, while the PVY ZT-5 WT strain, PVY-GFP, ChiVMV, and TVBMV were maintained on Y87 plants.

### Field survey of potyviral diseases

A total of 497 tobacco samples exhibiting symptoms typical of potyvirus infections, such as mosaic, vein banding, etching, and leaf vein necrosis, were collected from 13 tobacco-planting counties in Yunnan province, China, during 2016–2019. The counties include Shilin, Yiliang, Fuyuan, Luoping, Qilin, Shizong, Chengjiang, Eshan, Yimen, Yuanjiang, Zhaoyang, Zhenyuan, and Jingdong. These samples were screened by serological assays for the presence of PVY, ChiVMV and TVBMV, as detailed in the following section, and representative isolates were verified by DNA sequencing.

### Double antibody sandwich ELISA (DAS-ELISA)

DAS-ELISA were performed for screening of potyvirus infections in the field samples and assessment of potyvirus resistance at various time points after mechanical inoculation. In brief, leaf discs were homogenized in a 2-mL centrifuge tube containing 1 mL of 0.1 M phosphate-buffered saline (PBS, pH 7.2). After centrifugation at 5000 rpm for 10 min, the supernatant was used for DAS-ELISA assay using commercial kits for the detection of PVY (Agdia, IN, US; Cat# SRA20001/1000), TVBMV (Nano Diagnostics, CA, US; Cat# V250-R2) and ChiVMV (Nano Diagnostics; Cat# V246-R2), according to the manufacturer’s instruction. Each sample was repeated in triplicates and non-infected tobacco samples were used as negative controls. Absorbance values at 405 nm (OD_405_) was measured, and the values were adjusted by subtraction of buffer samples. A sample was considered positive if the OD_405_ value was two times higher than the mean value of negative control samples.

### Mechanical inoculation and agroinoculation

For mechanical transmission, 1 g of young leaf tissues from the infected plants was ground in 10 mL of 0.1 M PBS (pH 7.2) containing 0.5% celite to prepare virus inoculum. Crude leaf extracts were mechanically inoculated onto different tobacco varieties and genome-edited lines. Inoculated plants were grown at 25°C with 60% relative humidity under a 16-h light/8-h dark photoperiod and monitored for symptom appearance. Experiments were repeated three or four times, each with sample sizes ranging from 5 to 24 plants (biological replicates) per group to ensure statistical robustness.

The PVY ZT-5-derived infectious clones were individually electroporated into *Agrobacterium tumefaciens* strain EHA105. Agrobacteria were cultured in Luria–Bertani (LB) medium with 10 mM 2-(N-morpholino) ethanesulphonic acid (MES), 20 µM acetosyringone (AS) and antibiotics (50 µg/mL kanamycin and 25 µg/mL rifampicin). Suspensions of positive transformants were adjusted to a 0.7 optical density at 600 nm (OD_600_) in MES buffer (10 mM MgCl_2_, 10 mM MES, pH 5.6, 150 µM AS) and infiltrated into leaves of tobacco plants.

### Immunoblotting

Total protein samples were extracted from infected leaf tissues and separated by 12.5% SDS–PAGE, and transferred to nitrocellulose membranes. The membranes were first blotted with monoclonal antibodies raised against PVY CP (Song et al. 2016) and GFP (Abcam, UK; Cat# ab32146) and then with a HRP-conjugated goat anti-mouse secondary antibody (HuaAn, China). The dilutions of the CP, GFP, and secondary antibodies were 1:5,000, 1:8,000, and 1:10,000, respectively. Rubisco large subunits were stained with Ponceau S as protein loading controls.

### Cloning of VPg cistron and DNA sequencing

Total RNA was isolated from infected tobacco leaves using the TRIzol reagent (Invitrogen, Carlsbad, USA). The VPg coding sequence was amplified by reverse transcription-PCR (RT-PCR) using specific forward primer (5′-GACTTGGAAGAAGTCATTAGTGGC-3′) and reverse primer (5′-TCAGACGTTCCATATTCAACAGAC-3′) that anneal to the upstream and downstream sequence of the VPg cistron, respectively. The PCR products (510 bp) were gel-purified and cloned into the pMD-18T (TaKaRa, Dalian, China) through T-A cloning, and positive clones were randomly selected for Sanger sequencing from both directions. The deduced VPg amino acid sequences were analysed by multiple sequence alignment using the CLUSTALW method in the MEGA6 program (Tamura et al. 2013).

### Identification, cloning, and phylogenetic analysis of eIF4E isoforms

To identify tobacco eIF4E isoforms, we used eIF4E-S cDNA sequence (GenBank Accession No. KF155696) as a query to BLAST search the *N. tabacum* genome (https://solgenomics.net/organism/Nicotiana_tabacum/genome). Due to high sequence homologies existed between several members of the eIF4E family, we used several strategies to clone the complete coding sequence (CDS) of the 12 eIF4E members using total RNA extracted from tobacco K326 plants: (i) the CDSs of eIF4E2-S, eIFiso4E-T, nCBP1-S, nCBP1-T, nCBP2-S, and nCBP2-T were directly amplified by PCR, since their sequence are divergent enough to design specific primer pairs; (ii) for eIF4E1-S, eIF4E1a-T, eIF4E1h-T, eIF4E2-T, and eIFiso4E-S, the coding regions plus 5′ and/or 3′ UTR sequences were first amplified, followed by a nested PCR using internal primers; (iii) the eIF4E1b-T CDS was obtained by overlapping PCR using two sets of primer pair to ensure amplification specificity (Table S1). The obtained PCR products were cloned, sequenced from both directions. The complete CDS sequence of the 12 eIF4E members were deposited into GenBank under the accession numbers from MN896999 to MN897010 (Table S1). The deduced amino acid sequences of all eIF4E isoforms were used to construct the phylogenetic tree using the neighbor-joining method in MEGA 6 software. Maximum likelihood composite and complete deletion were used in the substitution model/method and gaps/missing data treatment, respectively. Bootstrap resampling (1000 replications) was used to estimate the statistical significance of each node.

### Yeast two-hybrid assays

The CDSs of individual eIF4E members were amplified using gene-specific primers and inserted into the pJP317 vector downstream of the activation domain (AD) between the *Eco*RI and *Xho*I restriction sites, while the coding sequences of the wild-type VPg or K105Q mutant were cloned into pYL302 downstream of the LexA DNA-binding domain (BD) between the *Sph*I and *Sac*I sites. The interactions between VPg and eIF4E members were analyzed using LexA yeast two-hybrid system (Clontech, CA, USA) according to manufacturer’s instruction. In brief, all constructs were transformed into the *Saccharomyces cerevisiae* strain SKY48 and grown on synthetic dropout medium lacking uracil, tryptophan, and histidine (SD-UTH^−^) at 30 °C for 72 h. Single colonies were picked, resuspended, and serially diluted. Aliquots of 5 μL culture were spotted onto SD-UTH^−^ medium or synthetic complete medium with 2% raffinose and 2% galactose and lacking uracil, tryptophan, histidine and leucine (SC-UTHL^−^) supplemented with 80 μg/mL X-gal for LacZ reporter gene activity analysis. Yeast cultures were incubated at 30°C for 3–5 days, and colony growth was monitored.

### Bimolecular fluorescence complementation (BiFC) assay

The coding sequences of selected eIF4E members were fused to the C-terminal half of the yellow fluorescent protein (YFP), and the VPg WT and K105Q mutant sequences were fused to the N-terminal half of the YFP. All constructs and empty vectors were transformed into *A. tumefaciens* strain EHA105 for transient expression in *N. benthamiana*. Agrobacterial cultures were grown, overnight, in YEP liquid medium supplemented with 10 mM MES (pH 5.6), 20 μM AS, and antibiotics (50 µg/mL kanamycin and 25 µg/mL rifampicin). Cultures reaching an optical density at 600 nm (OD_600_) of approximately 1.0 were centrifuged at 4,000 rpm for 10 min and washed twice with sterile distilled water. The bacterial suspensions were then resuspended in infiltration buffer (10 mM MES [pH 5.6], 10 mM MgCl_2_, and 100 μM AS), adjusted to an OD_600_ of 0.6, and mixed in equal proportions for co-infiltration. After a 3-h incubation, at room temperature, *Agrobacterium* suspensions were infiltrated into the leaves of 4–5-week-old *N. benthamiana* transgenic plants expressing red fluorescent protein fused with histone H2B (RFP-H2B) (Martin et al. 2009). YFP and RFP fluorescence was visualized and captured using an FV3000 confocal laser scanning microscope (Olympus, Japan). YFP and RFP fluorescence was separately excited at 514 and 561 nm, and images were processed with FV31S-SW (Olympus, Japan).

### CRISPR/Cas genome editing of *eIFiso4E* members

A CRISPR/Cas target site sequence in the first exon of both *eIFiso4E-T* and *eIFiso4E-S* genes were selected, and BLAST analysis verified the absence of off-target sites within the *N. tabacum* genome. Two complementary oligonucleotides corresponding to the selected target site (protospacer) sequence were synthesized, annealed, and ligated into the *Bsa*I-digested Cas9-PF vector as previously described (Liu et al. 2019). The resulting binary constructs were confirmed by DNA sequencing.

Leaf discs from 6-week-old Y87 and Y87^NIL^ plants were co-cultured with agrobacterial culture harboring the Cas9-PF vector and then cultured on induction medium (4.4 g/L Murashige and Skoog [MS] medium including vitamins, 30 g/L sucrose, 1 mg/L 6-benzylaminopurine, and 4 g/L phytagel, pH 5.8) with 50 μg/mL hygromycin B. Regenerated shoots were excised and transferred to a rooting medium (4.4 g/L MS medium including vitamins, 3 g/L sucrose, 7 g/L glucose, and 4 g/L phytagel, pH 5.8) and grown for approximately 3 weeks. Rooted plantlets (5–6 cm long) were acclimated and transplanted into the soil. Genomic DNA was extracted from stable transgenic tobacco plants and used for PCR amplification of the Cas9/sgRNA target regions using Q5 high-fidelity DNA polymerase (New England Biolabs). For the eIFiso4E-T editing target region, amplification was performed using specific primers: forward, 5′-GATTACCGGCCCAGTCTGTCATCAT-3′, and reverse, 5′-GGAACAAAATCCGAATTTATCAATAACT-3′. The eIFiso4E-S editing target region was amplified with a forward primer, 5′-CAATTCCATTACGCCTCTCCGTTCGCT-3′. PCR products (430 bp for eIFiso4E-T; 510 bp for eIFiso4E-S) were purified, cloned, and sequenced. Ten randomly selected colonies per target were analyzed to identify mutation types.

The Cas9-PF vector contains a visible purple marker, enabling efficient identification of transgene-free T_1_ plants through seedling color selection (Liu et al. 2019). All green T_1_ plants, indicating the absence of transgene elements, from *eIFiso4E-T* and/or *eIFiso4E-S* gene-edited T_0_ tobacco lines were screened for homozygous mutations and subsequent resistance analysis.

## Supplementary Information

Below is the link to the electronic supplementary material.

Supplementary file1 (DOCX 1124 KB)

## Data Availability

The data that support the findings of this study are available in this article from the corresponding author upon request.
