# Supplementary material for: Simultaneous knockout of multiple eukaryotic translation initiation factor 4E genes confers durable and broad-spectrum resistance to potyviruses in tobacco
Source: aBIOTECH. 2025 May 5;6(2):232–48. doi: 10.1007/s42994-025-00216-5 (PMC12238429; doi:10.1007/s42994-025-00216-5)
Supplement: Supplementary file 1 — Supplementary file1 (DOCX 1124 KB) [file 42994_2025_216_MOESM1_ESM.docx]

**Simultaneous knockout of multiple *eukaryotic translation initiation factor 4E* genes confers durable and b****road-spectrum resistance to potyviruses in tobacco**

Yong Liu^1, #^, Shuo Wang^2,3,4, #^, Danyang Zhao^2,3,4^, Chenglu Zhao^2,3,4^, Haiqin Yu^1^, Jianmin Zeng^1^, Zhijun Tong^1^, Cheng Yuan^1^, Zhenghe Li^2,3,4,*^, Changjun Huang^1,*^

^1^Yunnan Academy of Tobacco Agricultural Sciences, Key Laboratory of Tobacco Biotechnological Breeding, National Tobacco Genetic Engineering Research Center, Kunming 650021, China

^2^State Key Laboratory of Rice Biology, Institute of Biotechnology, Zhejiang University, Hangzhou 310058, China

^3^Ministry of Agriculture Key Laboratory of Molecular Biology of Crop Pathogens and Insect Pests, Zhejiang University, Hangzhou 310058, China

^4^Key Laboratory of Biology of Crop Pathogens and Insects of Zhejiang Province, Zhejiang University, Hangzhou 310058, China

^#^ These authors contributed equally to this work.

^*^ Correspondence: Zhenghe Li, [lizh@zju.edu.cn](mailto:lizh@zju.edu.cn); Changjun Huang, cjhuang@zju.edu.cn

**Supplementary Information**

**Table of Contents**

- **Supplementary Figures**

**Fig. S1** Alignment of partial amino acid sequences of the VPg protein from PVY variants propagated in *N. tabacum* genotypes Hongda (*Va*) and TN86 (*va*).

**Fig. S2** Alignment of nucleotide sequences of the *eIFiso4E-S* and *eIFiso4E-T* coding domains (CDS).

- **Supplementary Tables**

**Table S1** Primers used for amplifying the cDNA of the eIF4e family members

**
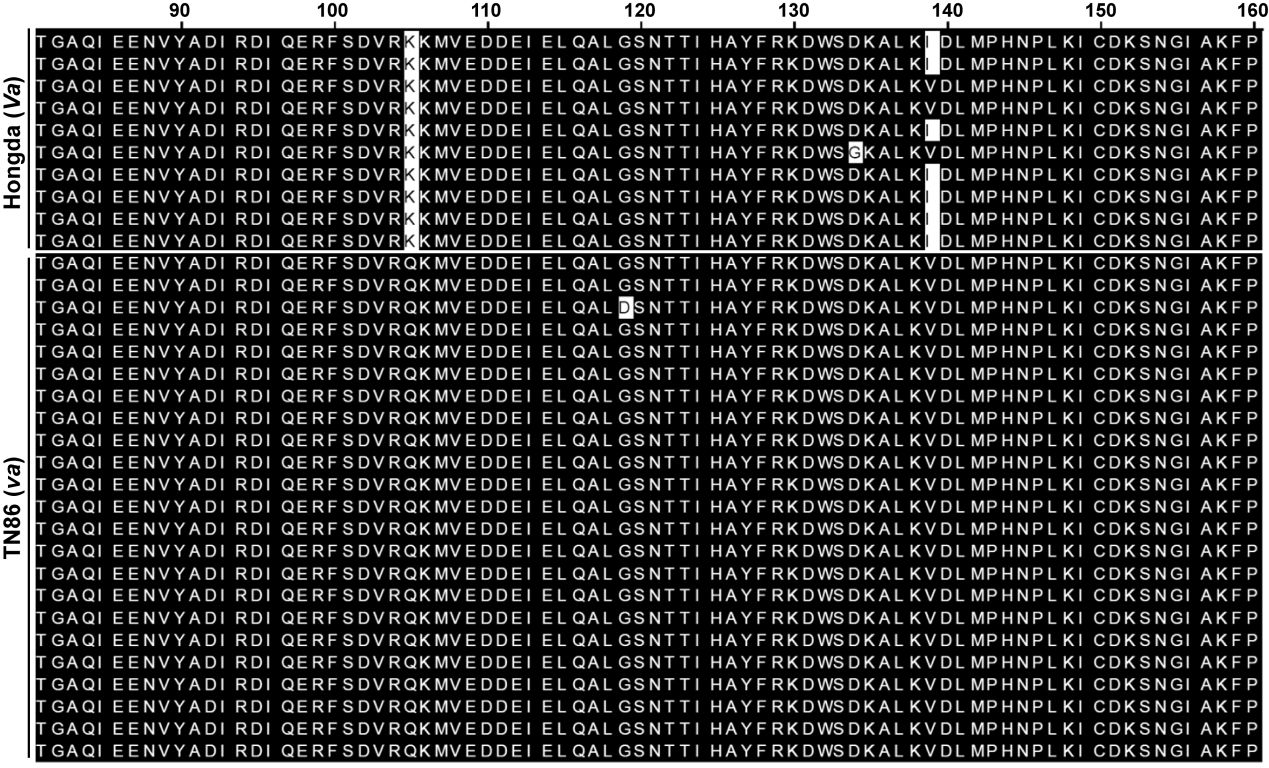
**

**Fig. S1** Alignment of partial amino acid sequences of the VPg protein from PVY variants propagated in *N. tabacum* genotypes Hongda (*Va*) and TN86 (*va*). The alignment focuses on the amino acid region spanning positions 81 to 160. A total of twenty-three VPg clones derived from TN86 and ten clones from Hongda are included in the alignment. Identical residues are highlighted in black, and the numbering at the top corresponds to the position of the residues.

**
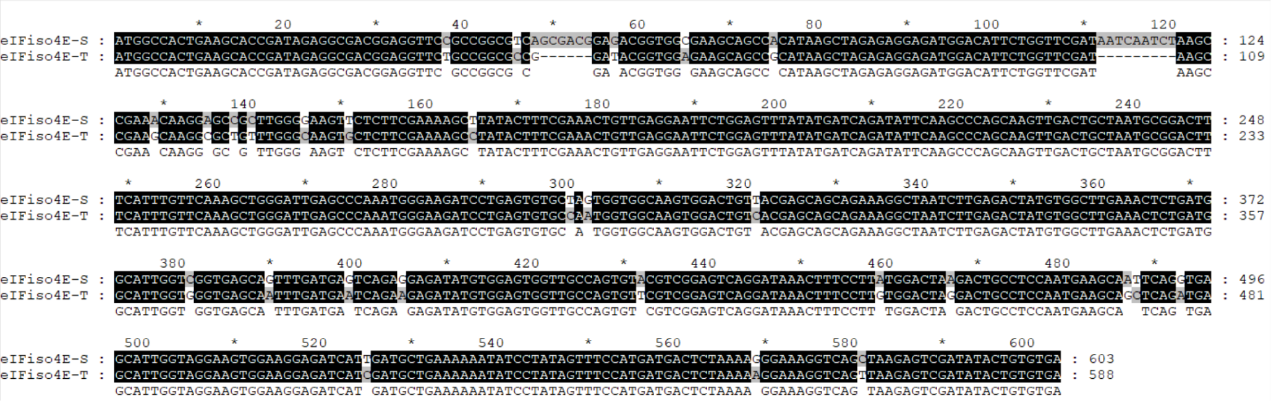
**

**Fig. S2** Alignment of nucleotide sequences of the *eIFiso4E-S* and *eIFiso4E-T* coding domains (CDS). The alignment displays the full-length sequences of both CDS, with identical residues highlighted in black. Nucleotide positions are indicated at the top and right.

**Table S1** Primers used for amplifying the cDNA of the *eIF4e* family members

| Gene | GenBank No. | Forward primer (5′→3′) | Reverse primer (5′→3′) | Notes |
| --- | --- | --- | --- | --- |
| eIF4E1-S | MN896999 | TTCCTACTAAAATCTATAACTGAGTACA | CTATACTGTATAACGATTCTTGGCACCTC | Amplify 5′ UTR and CDS |
| eIF4E1a-T | MN897000 | AATCTATTACTAAAAAGCAAGCTACAACC | CTATACCGTATAACGATTCTTGGCACCTTT | Amplify 5′ UTR and CDS |
| eIF4E1b-T | MN897001 | ATGGCAGGGGAAGCGAAAATGGCA | CTTCACAATGCTAACCGTGTATAGCCAGCT | Amplify the 5′ end (1-450) of CDS |
|  |  | AGCTGGCTATACACGGTTAGCATTGTGAAG | CTATACTGTATAGCGATACTTGGCACCTC | Amplify the 3′ end (421-552) of CDS |
| eIF4E1h-T | MN897010 | TAGCTAAAAGCAAGCAAAGTACATC | TCCAGTCCAGATTCTGTTTCTTG | Amplify 5′ UTR, CDS, and 3′ UTR |
| eIF4E2-S | MN897002 | ATGGTTGATGAAGTAGAGAAACCGGT | CTACACAGAATAACGATTCTTGGCAG | Amplify complete CDS |
| eIF4E2-T | MN897003 | CCTCAATATATACATATATATGAATACAGTATCG | CTGTGAATACCAATGTAAGAGGC | Amplify 5′ UTR, CDS, and 3′ UTR |
| eIFiso4E-S | MN897005 | TTCCATTACGCCTCTCCG | TGCTAATCGTACAATTTTCAGTATTGG | Amplify 5′ UTR, CDS, and 3′ UTR |
| eIFiso4E-T | MN897004 | ATGGCCACTGAAGCACCGATAGAG | TCACACAGTATATCGACTCTTAACTG | Amplify complete CDS |
| nCBP1-S | MN897006 | ATGGAAGTGACAGCTGAGAAGAGAGAATTG | TTATCCTCTTATCCAAGTATTGCGGTATG | Amplify complete CDS |
| nCBP1-T | MN897007 | ATGGAAGTGACAGCTGAGAAGAGAGAATCA | TTATCCTCTTATCCAAGTATTGCGGTATG | Amplify complete CDS |
| nCBP2-S | MN897008 | ATGGAAGTGACGGGGGAGAAGA | CTATCCTCTCAGCCAAGTATTGCA | Amplify complete CDS |
| nCBP2-T | MN897009 | ATGGAAGTGACGGGGGAGAAGA | TTATCCTCTCAGCCAAGTATTGCG | Amplify complete CDS |
